# Feasibility of conducting a pilot randomized control trial of a psycho-education intervention in patients with a first episode psychosis in Uganda—A study protocol

**DOI:** 10.1371/journal.pone.0268493

**Published:** 2022-07-29

**Authors:** Dickens Akena, Aggrey Semeere, Philippa Kadama, Emmanuel K. Mwesiga, Juliet Nakku, Noeline Nakasujja

**Affiliations:** 1 Department of Psychiatry, Makerere University College of Health Sciences, Kampala, Uganda; 2 Infectious Disease Institute, Makerere University College of Health Sciences, Kampala, Uganda; 3 Butabika National Mental referral Hospital, Kampala, Uganda; Public Library of Science, UNITED STATES

## Abstract

**Background:**

Psychotic disorders contribute to significant morbidity and mortality partly due to the chronicity of the illness and high relapse rates. Delivering psycho-education messages about disease etiology, their signs and symptoms and the benefits of treatment adherence have been shown to improve clinical outcomes among individuals with psychoses. However, little has been done to examine the feasibility and efficacy of this intervention in low resourced settings.

**Objective:**

Our primary objective will be to determine the feasibility of recruiting and retaining patients with a first episode psychosis (FEP) and for the secondary objective, we will determine the preliminary efficacy of psycho-education on illness self-management, stigma, adherence to medications and symptom severity.

**Hypothesis:**

We hypothesize that (i) we will recruit 70% of eligible participants and accrue a sample size of 80 over 20-weeks, retaining 80% of the sample size for 24 weeks, (ii) the intervention will lead to improvement in clinical outcomes (described above).

**Methods:**

We will recruit 80 adult patients who have been diagnosed with a FEP, received antipsychotic medication at Butabika Hospital and reside within 21km from the Hospital. Trained village health team (VHTs) members will deliver 6 psycho-education sessions to 40 participants and their family members (intervention arm). Participants in the control arm (n = 40) will receive routine care. We will document how feasible it will be to recruit and retain participants over 24 weeks and document the preliminary efficacy of the intervention on illness self-management, stigma, adherence to medications and severity of symptoms.

**Data analysis:**

We will document the proportion of participants who consent and get recruited, the proportion of those who will get retained and reasons for drop out. We will conduct an intention to treat analysis comparing the groups at weeks 4, 12, 24 and assess the effect of the intervention on the clinical outcomes (described above). We will use the Bonferroni approach to correct for multiple comparisons.

**Trial registration:**

Clinical trials.gov registration number: NCT 04602585.

## Introduction

Psychotic disorders significantly contribute to the global burden of disease and disability, with sub-Saharan Africa (SSA) bearing a huge brunt of this burden [1–3]. For this study, we operationally defined psychosis as any of schizophrenia or schizophrenia spectrum disorder, brief psychotic episode, or bipolar affective disorders. Psychotic disorders have been shown to predict poor quality of life [4, 5], increased health care costs [6, 7], higher mortality mainly due to suicide, accidents, and comorbid infectious diseases [8–10] as well as non-infectious diseases like hypertension, obesity and diabetes mellitus [11–13] Literature shows a high prevalence of psychiatric illnesses such as depression, anxiety and substance misuse disorders [14–16] among patients with psychosis.

Due to the high burden of psychotic disorders, several scholars have recommended that it is identified and treated with antipsychotic medications—the main form of treatment. However, almost of half of individuals with psychoses relapse within 2–3 years of commencing treatment mainly due to poor medication adherence [17], yet long-term adherence is essential for ensuring the achievement of social and occupational functioning [18, 19]. The addition of interventions (adjunct) that are geared towards improving adherence to medication and stigma reduction have been shown to improve outcomes (retention in care and reduction in relapse) in persons taking antipsychotic medications [20, 21]. Psycho-education is among the most used adjunct intervention for individuals with psychosis—during these interventions, structured messages describing the aetiology of psychoses, their signs and symptoms, and the benefits of treatment are delivered. Most psycho-education studies have been conducted in high income countries (HIC) [20, 21] with little having been done in low resourced settings such as Uganda.

A number of factors makes it inappropriate to extrapolate findings from HIC to low resourced settings. First, inadequate funding for mental health interventions in resource constrained settings such as Uganda [22] means little may be done with regards to delivery of psycho-education—such activities may be considered extra workload. In the majority of low resourced settings, a prescription of antipsychotics (with little in the way of providing psycho-education) is the standard of care. Moreover, variations in clinical or symptom presentation, conceptualization of psychoses (negative explanatory models of psychosis and stigma), and disease severity in some ethnic groups across multiple populations in the world [21, 23–27] may dictate the way individuals accept treatment and ultimately influence the type of treatment that they receive [28]. Furthermore, the heterogeneous nature of psychoses including differences in responses to antipsychotics [25, 29–33] makes it inappropriate to extrapolate findings about the efficacy of psycho-education interventions from HIC to sub-Saharan Africa (SSA). Studies that examine culturally appropriate relapse intervention techniques are urgently needed.

### The problem

The high rates of relapse among individuals with psychoses only leads to higher rates of mortality (through suicide) and morbidity. With each relapse individuals will respond poorly to medications, stay longer [34] in hospital stay and lead unhealthy lifestyles—this will limit their longevity making it difficult to achieve multiple development milestones [35–37]. The need for relapse prevention interventions like psycho-education cannot be over emphasized. However the efficacy of adjunct therapies such as psycho-education remains to be known, as well as how feasible it is to recruit and retain participants for such interventions.

We propose to document the feasibility of recruiting and retaining persons with a first episode psychosis (FEP) and preliminary efficacy of a psycho-education intervention that will be delivered by village health team members (VHT). The operational definitions of a FEP varies widely [38], but for this study, individuals will be considered to have a FEP if they have (a) experienced psychotic symptoms for the very first time in their lives, (b) experienced psychotic symptoms before, but are accessing psychiatric care (antipsychotic medications) for the very first time in their lives at the study site, or (c) if already on antipsychotics or used antipsychotic medications for no longer than 6 weeks and (d) if the psychosis is not primarily a result of substance use disorders or a medical illness like HIV.

### Objective

Our primary objective will be to determine the feasibility of recruiting and retaining patients with a FEP and for the secondary objective, we will determine the preliminary efficacy of psycho-education on illness self-management, stigma, adherence to medications and symptom severity.

### Hypothesis

We hypothesize that (i) we will recruit 70% of eligible participants and accrue a sample size of 80 over 20-weeks, retaining 80% of the sample size for 24 weeks, (ii) the intervention will lead to improvement in clinical outcomes in patients with FEP (see above).

### The VHTs in the health care system

Village Health Teams (VHTs) were established by the Ugandan Ministry of Health in 2001 to empower communities to take part in the decisions that affect their health, mobilize communities for health programs, and strengthen the delivery of health services at house-hold level. Each VHT serves 25–30 households with a minimum of one third of VHTs per village being women. Potential VHT members may already be community health workers, traditional birth attendants, medication distributors or other individuals who are already providing adjunct care within the community. VHTs are chosen by popular vote and engage with members of the community through home visits and community dialogue with targeted messages. At least 4/10 people who reach the health centre are referred by the VHTs as per MOH reports of 2015.

In 2017, the MOH of Uganda trained VHTs in delivery of some aspects of mental health care using the WHO mhGAP—however, the feasibility of engaging them to deliver interventions for persons with psychosis is yet to be examined. For the proposed study, we will choose from among the MOH trained VHTs and train those who may have missed the MOH mental health training of 2017 to deliver psycho-education using materials described below.

### The psycho-education materials

The psycho-education materials consist of a combination of text and pictures that we developed through consultation with mental health specialists, patients and their caretakers. A number of psycho-education materials were developed for common mental disorders and psychosis in our earlier study [39]. We conducted qualitative interviews during the development of the documents. The psycho-education materials are being used for the very first time in the Ugandan setting.

Topics in the booklet (attached as S1 File) include information about i) The signs and symptoms of psychosis, ii) the challenges faced by people with psychosis including disruption in social and occupational functioning, iii) potential causes of psychosis (biological, substance use, trauma, stress), iv) the treatment options for psychosis(antipsychotics and adjunct psychosocial therapies) v) rationale for treating psychosis (reduction in relapse, improvement in ability to function), and vi) the myths about psychosis (dispelling stigmatizing undertones and challenging non-medical explanatory models of mental illness).

Each of the 6 topics lasts about an hour. To ensure standardization (all participants receive the same information), we converted the information into audio (English and Luganda). The VHT will play the audio as participants listen in and will then provide them with the written text for their future reference.

## Methods

### Study design

A pilot RCT to examine feasibility of recruitment and retention and preliminary efficacy of our intervention.

### Study setting

The study will be conducted at the Butabika National Mental Referral Hospital in Kampala, a 600 bed hospital located 13km east of Kampala city. The hospital has 3 acute admission wards, 3 convalescent wards (housing patients with less acute symptoms and ready for discharge), one male and one female sick ward (where individuals with physical illnesses are admitted), an alcohol and drug unit, a child and adolescent unit and a private wing. It has a medical out-patient that provides a service to HIV/AIDS patients, a dental clinic and a general out-patients’ clinic. The out-patient clinics operates weekdays from 9 am–5 pm. Each of the units (in-patient and out-patient) is run by team of psychiatrists, medical officers, psychiatric clinical officers (physician assistant with 3-year psychiatry training), psychiatric nurses, clinical psychologists and psychiatric social workers. We will recruit participants from the convalescent wards (clinically stable patients awaiting discharge).

#### Standard of care

At Butabika Hospital, patients with acute illnesses are admitted, treated with psychotropic medications and discharged usually over a 2-3-week period. The patients return to pick up their medications and get assessed by the clinician on duty. The clinician on duty could be a psychiatrist, medical officer or psychiatric clinical officer (an assistant physician equivalent). The patients receive little by way of psycho-education or any other form of psychological therapy—this is primarily due to the large numbers and few health care providers.

### Eligibility criteria

Individuals will be eligible if they have been diagnosed with a FEP and considered (by the attending clinician) to have demonstrable resolution of active symptoms following the use of antipsychotic medications and deemed clinically stable for a discharge. The FEP diagnosis will be confirmed through an interview conducted by a trained bachelors level research assistant (RA) using the Mini International Neuropsychiatric Instrument (M.I.N.I) [40]. Participants (adults ≥18 years) will be required to provide written informed consent and should reside within a 21 km radius from the hospital.

#### Exclusion criteria

Individuals will be excluded if they present with a psychosis secondary to a medical or substance use disorder.

### Sample size estimation

Being a study assessing feasibility of recruitment as a primary outcome, we have not conduced a formal sample size and power calculation. Rather, we have considered that recruiting 70% of potentially eligible participants over a 20-week period and retaining 80% of recruited participants will provide us with adequate data that will be needed in recruiting participants for a powered RCT.

In 2018 [41], a total of 43,870 individuals accessed care at the hospital, 60% (26,322) of them were diagnosed with psychotic disorders, approximately 20% (5,264) of the 26,322 patients with psychosis presented for the first time (potential cases of FEP). Thus, the number of participants who are eligible for recruitment is approximate 440 per month. Close to 20% of the Ugandan population lives within the central districts from where we intend to recruit participants—we anticipate having 88 patients (with psychosis) eligible for recruitment per week. However, a significant proportion of patients present with a psychosis resulting from substance use. We anticipate that approximately 40% of the 88 patients would be eligible as per criteria described below. Thus, we will have approximately 32 participants eligible for recruitment per week with a recruitment target of 24 of the 32 participants per week. We will document what proportion of this figure is attained.

With a sample size of 80 participants, we will be able to conduct preliminary analyses to detect modest significant differences (5% increase in mean scores) in illness self-management and adherence as well as a 5% reduction in mean stigma and symptom severity. We will use this data to generate an effect size that can be used in a sample size estimation of a powered RCT.

### Data collection methods

#### Training

RAs and VHTs will each undergo 4-day’s training in the study procedures.

#### Random sequence allocation

We will use a simple random technique to randomize participants. Random numbers that determine which study arm the participant will be in will be generated by an independent statistician who will not be part of the study team.

#### Data collection

The health care providers based in the wards from where we will collect data will be informed about the study. They will identify patients who may be eligible and refer them to the RA for a second round of eligibility screening. The RAs shall provide potential participants with information about the study, obtain written informed consent, then administer the University of California, San Diego Brief Assessment of Capacity to Consent (UBACC) [42] to assess participant comprehension of the consent process.

RA’s will administer a) standardized questionnaire to collect demographic and clinical variables (age, gender, address, education, date of symptom onset) from the patients. We will collect demographic information in the form of age, gender, address and education from care givers. b) Feasibility measures (number of eligible participants who accept to enroll in the study, number of participants who are retained in care over study duration, number of participants who adhere to prescribed intervention, duration of recruitment of participants, reasons for decline to enroll in the study as well as reasons for dropping out of the study), c) MINI to confirm a diagnosis, d) Young Mania Rating Scale (YMRS) [43] for individuals with bipolar illness or Positive and Negative Symptoms of Schizophrenia Scale (PANSS) [44] for individuals with schizophrenia spectrum disorders to assess symptom severity e) World Health Organization Disability Assessment Schedule, f) Internalized Stigma of Mental Illness scale to assess for stigma, g) a medication adherence measure to assess for adherence h) Illness Management and Recovery (IMR) [45] scale to document the ability of patients to manage their illness and (i) care giver burden using the short version of the burden scale for care givers (BSFC) [46].

#### Blinding participants and RA to the study arm

Participants will be randomly assigned to the study arms. The RA and participants will all be blinded to the study arm they will be allocated before consent. VHTs will deliver 6 psycho-education sessions (1 per month) to participants in intervention arm and their family members at the participant’s residence. The PI and RAs will sit in some of the sessions during the first 2–3 sessions of data collection to ensure fidelity to the manual. We will conduct 5 exit focus group discussions with patients and family members to assess acceptability to the use of the manual. Participants in the control arm will get usual treatment. RA’s will schedule participants from both arms for an assessment using the same instruments at weeks 4, 12 and 24 at the clinic. We will have a separate set of RA’s for the intervention arm and another for the control arm to ensure blinding of the RA.

### Participant identification and recruitment

#### Identification

Health care workers in the different wards and out-patient departments of Butabika Hospital will be informed about the study. Trained RA shall liaise with the clinicians to identify potential participants for recruitment at the time of admission to the wards. RA’s will then assess participants who are due for discharge (patients with a clinical response to medications) for possible enrollment and provide them with information about the study. Patients who access care at the out-patient clinic (who may not be admitted) but are eligible will also be approached by the RA for enrollment.

#### The informed consent procedure

The RA’s will invite potential participants to take part in the study and obtain written informed consent. During the consenting process, the purpose of the study will be described further, the procedures will be explained, the benefits and risks of taking part in the study will be outlined. Upon demonstrating understanding and being given a chance to ask questions, the potential participant will then provide a witnessed, signed or thumb print consent. Caregivers will also be required to provide written informed consent, since we will collect demographic information from them.

#### Assessing the capacity to consent

We will administer the University of California, San Diego Brief Assessment of Capacity to Consent (UBACC) [42] instrument to assess whether the participants have understood the consent process. The UBACC will be translated into Luganda, the commonly spoken local language at the study site. The UBACC is a 10-item scale comprised of 3 factors that evaluate understanding, appreciation and reasoning. It has been used in the Ugandan setting for the Neuro-GAP project, although it is yet to be culturally adapted for use in these settings. A score of less than 14.5 on three separate occasions indicates that the participant has not understood what the study is all about. Such participants will not be recruited but will be given a chance to return at a later date for recruitment (within a week). We will record the number of participants who fail the UBACC at baseline and can’t be recruited and those who do so after being invited a week later.

We will ask participants to provide us with their demographic information (age, gender and education level) and examine whether there are significant differences between participants who are able to consent and those who are not. Participation in this study is completely voluntary. The patient has the right to withdraw at any time during the study, including at follow-up. We will document the reasons for withdrawal. Each interview is anticipated to last a minimum of 120 minutes—there will be 2–3 breaks at 40 minute intervals in between the interviews.

### Study measurements

Trained RA will administer the following standardized questionnaires to all participants (see Table 1 below). All study questionnaires will be translated into Luganda, the commonly spoken local language at the study site.

- We will document the number of participants who agree to provide written informed consent; we will record the reasons for declining to consent. We will use standardized forms to record the number of participants who will return to the clinic for monthly review as well as those who will complete the psycho-education sessions (intervention arm only).
- Demographic and clinical parameters: We will (a) document the age, gender, physical address, contact information, marital and employment status, education level, date/month/year of onset of current illness. We will document whether the participant lives within a catchment area of ministry of health supported VHT; we will separately contact the VHT and get their details. We will also request for information from the next of kin for future contact in the event of a loss to follow-up. (b) we will document the age of onset of symptoms, duration of illness before accessing hospital care (acute if it is ≤ 6 months of onset and chronic if it is ≥2 years), whether the participant has received prior treatment for the psychosis (traditional or faith healers), whether the patient had a say in the choice of antipsychotics that was prescribed to them.
- We will administer the UBACC [42] to assess participant’s capacity to provide informed consent.
- The M.I.N.I 7.0.2 [40] psychosis, depression, bipolar affective disorders, substance use disorder, PTSD and generalized anxiety modules will be used to confirm the presence of a psychoses, and other CMD. The MINI has been used in multiple Ugandan study settings including the Neuro-GAP project, although it is yet to be validated for use in these settings
- Symptom severity assessed using the YMRS [43] or PANSS [44]. Both the YMRS and PANSS have been used in Ugandan study settings although it is yet to be validated for use in these settings
- The presence of medication side effects will be assessed using the modified version of the Glasgow Antipsychotic Side Effects Scale (GASS)
- The World Health Organization Disability Assessment Schedule Version 2 (WHODAS 2.0) will be used to assess the level of social and occupational functioning of participants. There is limited data about the use of the WHODAS in Ugandan populations.
- Adherence to antipsychotics will be measured using the medication adherence rating scale (MARS) for psychosis [47].
- We will document mortality from any causes in the participants using the WHO verbal autopsy scale.
- illness management ability will be assessed using the IMR [45].
- The care giver burden will be measured using the short version of the burden scale for care givers (BSFC) [46].

**Table 1:** Summary of the study instruments to be administered at baseline and follow-up.

Instrument	Baseline	Follow-up
Consent forms, UBACC(administered a day earlier)		
Demographic parameters	Yes	No
UBACC	Yes	No
MINI	Yes	Yes
Physical exam	Yes	Yes
Symptom severity using the YMRS/PANSS	Yes	Yes
GASS	Yes	Yes
WHODAS 2.0	No	Yes
Medication adherence using the MARS	No	Yes
WHO Audit to assess all course mortality	No	Yes
Illness Management and recovery	No	Yes
BSFC (participant’s care givers only)	Yes	Yes

### Testing the data collection process

We will collect data among 5 participants to help us (i) assess for clarity of study questionnaires (ii) identify any barriers to data collection and study implementation and (iii) figure out ways of circumventing the barriers to study implementation if any. The data from these 5 participants will not be included in the final analysis.

### The intervention

After discharge, the program manager will link the participants with a VHT nearest to them. The participants (randomized to the intervention arm) will be informed during the consent procedure that they will undergo 6 psycho-education sessions (1 per month) together with a family member at the participant’s residence. The VHTs and participants will meet and schedule appointments for the next engagements. This shall be done on a case by case basis. Some psycho-education sessions could take place in the patient’s residence, others in the nearest public space (school or church or mosque compounds). We will document where the majority of these sessions happen. This will help us document feasibility. The PI and RAs will sit in some of the sessions during the pilot phase of data collection to ensure fidelity to the manual.

### Follow-up

Participant Follow-up: We shall collect the same data as at baseline from participants at month 3 then every six months. If the participant accesses health care and we are not aware, we will review their medical records and abstract information collected during the course of routine care (where they exist). However, all attempts will be made to always collect data directly from the participants.

### Medical record review

There is a possibility that participants in the control arm will return to access care at the facility and be missed by the RA. They could also be admitted to the facility due to other health complications. We will review participant’ medical charts and abstract information about any admission to the hospital, duration of stay, laboratory parameters, and any other recorded complications.

### Adverse event reporting during participant follow-up

We anticipate that this project will have minimal adverse events that are directly related to the study. However, if we observe any adverse events as a result of the use of prescribed medications during clinical care or loss of privacy/confidentiality, then we will report it promptly to the relevant regulatory bodies per requirement. Confidentiality could be broken in the event that the RA gets information related to the following: a) participant is suicidal, b) participant threatens to commit a homicide, and c) participants reports a sexual abuse to themselves or other parties. The RA will immediately inform the PI about such, and appropriate action will be taken including reporting such cases to the administration of Butabika Hospital.

#### Potential risks

There is a potential risk of developing severe psychological distress during the interviews as a result of answering questions that are deemed private by the participant. RA will be trained to identify any of such distress, and the interview will be terminated. Participants may be asked to continue with the interview only if they feel like doing so. Such adversities will be reported to the PI and SOMREC. There is also a risk of having information about participants made available in the public domain. We will guard against this by having all identifying information of the patients locked away in file cabinets and password locked computers. The risk to loss of information is minimal.

#### Benefits

There are no direct monetary benefits to be gained by the individuals. However, participants will receive regular assessments for their symptoms for the study period. Those in the intervention arm will receive psycho-education sessions. Furthermore, participating in this study will also help scientists to learn more about what predicts relapse in patients with psychosis.

#### Community tracing

If a participant has not appeared for a clinic visit on their scheduled clinic appointment, we will contact them or their appointed person through telephone. If neither the patient nor the contact person can be reached by phone three months from the last date of their scheduled appointment, we will make active attempts to trace the participant at their residence by liaising with the VHT based in the same location. If the participant can’t be traced at their place of residence, we will consider them as a potential loss to follow-up. There exists several village health team members who provide care to non-mental health clients. We will utilize their knowledge about the village to identify individuals in the cohort.

### Data analysis plans

We will report the duration of recruiting participants and the proportion of eligible patients who get recruited. We will compare the demographic parameters (age, gender, education status) as well as disease severity. We will compare the proportion of participants who are retained in the study at week 4, 12 and 24 as well as mean differences in adherence, stigma, illness management ability and symptom severity (PANSS or YMRS) scores between the intervention and control arms. We will use the Bonferroni approach to correct for multiple comparisons.

### Ethics and dissemination plans

This work received ethical permission from the Makerere University School of Medicine Research and Ethics Committee IRB approval number (**Rec Ref 2020–161**). Uganda National Council for Science and Technology (**HS 1026ES**).

#### Ethical considerations

We will have a two-layered consent process. (i) Trained RAs will read the consent form to the participant, then (ii) administer the UBACC to ensure that participants have fully understood the rationale for conducting the study and that they are participating well aware of their rights as participants.

A few ethical considerations are worth pointing out—beyond the distress that participants may experience, this will be a minimal risk study. To minimize the risk of loss of confidentiality and privacy, only the investigators will have access to the study records and test results and the link between personal identifying information and study data. No individual identities will be used in any reports or publications associated with the data from this study. All softcopies of the data will be stored in password locked computers. Hard copies of the questionnaires will be stored in locked file cabinets at the study offices in Butabika National Referral Hospital.

#### Dissemination plans

A manuscript will be prepared from these findings and submitted to peer reviewed journal for publication. Findings will also be presented at local and international conferences. We will hold a dissemination workshop and provide results to the patients who have participated in the pilot phase as well as other stakeholders to whom these findings are important in shaping policy and practice.

## Supporting information

S1 Checklist SPIRIT 2013 checklist: Recommended items to address in a clinical trial protocol and related documents*. (DOC)

S1 File English Psycho-education Booklet, response to the editors and reviewers. (PDF)

S2 File (DOCX)
