# Supplementary material for: Feasibility of conducting a pilot randomized control trial of a psycho-education intervention in patients with a first episode psychosis in Uganda—A study protocol
Source: PLoS One. 2022 Jul 29;17(7):e0268493. doi: 10.1371/journal.pone.0268493 (PMC9337703; doi:10.1371/journal.pone.0268493)
Supplement: S1 Checklist — (DOC) [file pone.0268493.s001.doc]

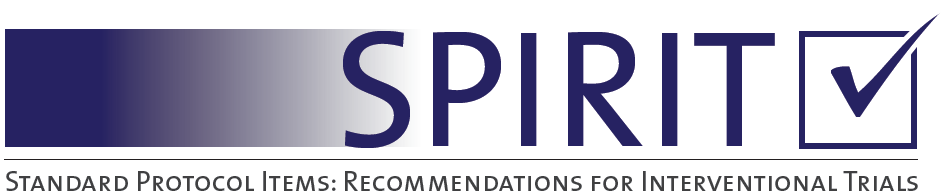


SPIRIT 2013 Checklist: Recommended items to address in a clinical trial protocol and related documents*

| Section/item | ItemNo | Description |
| --- | --- | --- |
| **Administrative information** | | |
| Title | 1 | The effect of psycho-education on clinical outcomes among patients with a first episode psychosis in central Uganda - A pilot randomized control trial.  (included in the title page) |
| Trial registration | 2a | Trials.gov. NCT04602585( included in the abstract) |
|  |  |  |
| Protocol version | 3 | Final version as of 09^th^ November 2020 |
| Funding | 4 | The study received limited funding from Makerere University Research and Innovations Fund (MAK/DVCFA/113/20). ( included in the declarations page) |
| Roles and responsibilities | 5a | Names, affiliations, and roles of protocol contributors .(The names of the authors and their affiliations are included in the title page while their roles and contributions are in the declarations page) |
|  | 5b | Name and contact information for the trial sponsor (The name of the contact person is included in the title page) |
|  | 5c | Role of study sponsor and funders, if any, in study design; collection, management, analysis, and interpretation of data; writing of the report; and the decision to submit the report for publication, including whether they will have ultimate authority over any of these activities (This information is included in the declarations page) |
|  |  |  |
| Introduction |  |  |
| Background and rationale | 6a | Description of research question and justification for undertaking the trial, including summary of relevant studies (published and unpublished) examining benefits and harms for each intervention (The introduction on page 1 captures this) |
|  | 6b | Explanation for choice of comparators (page 1) |
| Objectives | 7 | Specific objectives or hypotheses( the specific objective is on page 1 and the hypothesis on page 4) |
| Trial design | 8 | Description of trial design including type of trial (eg, parallel group, crossover, factorial, single group), allocation ratio, and framework (eg, superiority, equivalence, noninferiority, exploratory) ( the information about the design, a pilot randomized trial is on page 2) |
| Methods: Participants, interventions, and outcomes | | |
| Study setting | 9 | Description of study settings (eg, community clinic, academic hospital) and list of countries where data will be collected. Reference to where list of study sites can be obtained (the study setting is described on page 2) |
| Eligibility criteria | 10 | Inclusion and exclusion criteria for participants. If applicable, eligibility criteria for study centres and individuals who will perform the interventions (eg, surgeons, psychotherapists) (these criteria are included on pages 1-2) |
| Interventions | 11a | Interventions for each group with sufficient detail to allow replication, including how and when they will be administered (included on page 3) |
|  | 11b | Criteria for discontinuing or modifying allocated interventions for a given trial participant (eg, drug dose change in response to harms, participant request, or improving/worsening disease) (Not applicable) |
|  | 11c | Strategies to improve adherence to intervention protocols, and any procedures for monitoring adherence (eg, drug tablet return, laboratory tests) (Not applicable) |
|  | 11d | Relevant concomitant care and interventions that are permitted or prohibited during the trial (this is included as access to other services on page 5) |
| Outcomes | 12 | Primary, secondary, and other outcomes, including the specific measurement variable (eg, systolic blood pressure), analysis metric (eg, change from baseline, final value, time to event), method of aggregation (eg, median, proportion), and time point for each outcome. Explanation of the clinical relevance of chosen efficacy and harm outcomes is strongly recommended (the outcomes are described on pages 5-6) |
| Participant timeline | 13 | Time schedule of enrolment, interventions (including any run-ins and washouts), assessments, and visits for participants. A schematic diagram is highly recommended (see Figure) (this information is available on page 3) |
| Sample size | 14 | Estimated number of participants needed to achieve study objectives and how it was determined, including clinical and statistical assumptions supporting any sample size calculations (sample size calculation is on page 4) |
| Recruitment | 15 | Strategies for achieving adequate participant enrolment to reach target sample size (pages 4-5) |
| **Methods: Assignment of interventions (for controlled trials)** | | |
| Allocation: |  |  |
| Sequence generation | 16a | Method of generating the allocation sequence (eg, computer-generated random numbers), and list of any factors for stratification. To reduce predictability of a random sequence, details of any planned restriction (eg, blocking) should be provided in a separate document that is unavailable to those who enrol participants or assign interventions (this information is on page 3) |
| Allocation concealment mechanism | 16b | Mechanism of implementing the allocation sequence (eg, central telephone; sequentially numbered, opaque, sealed envelopes), describing any steps to conceal the sequence until interventions are assigned (this information is on page 3) |
| Implementation | 16c | Who will generate the allocation sequence, who will enrol participants, and who will assign participants to interventions (this information is on page 3) |
| Blinding (masking) | 17a | Who will be blinded after assignment to interventions (eg, trial participants, care providers, outcome assessors, data analysts), and how (this information is on page 3) |
|  | 17b | If blinded, circumstances under which unblinding is permissible, and procedure for revealing a participant’s allocated intervention during the trial (this information is on page 3) |
| **Methods: Data collection, management, and analysis** | | |
| Data collection methods | 18a | Plans for assessment and collection of outcome, baseline, and other trial data, including any related processes to promote data quality (eg, duplicate measurements, training of assessors) and a description of study instruments (eg, questionnaires, laboratory tests) along with their reliability and validity, if known. Reference to where data collection forms can be found, if not in the protocol (this information is on pages 3-4) |
|  | 18b | Plans to promote participant retention and complete follow-up, including list of any outcome data to be collected for participants who discontinue or deviate from intervention protocols (not applicable) |
| Data management | 19 | Plans for data entry, coding, security, and storage, including any related processes to promote data quality (eg, double data entry; range checks for data values). Reference to where details of data management procedures can be found, if not in the protocol (this information is on pages 3-4) |
| Statistical methods | 20a | Statistical methods for analysing primary and secondary outcomes. Reference to where other details of the statistical analysis plan can be found, if not in the protocol (this information is on pages 3-4) |
|  | 20b | Methods for any additional analyses (eg, subgroup and adjusted analyses) (this information is on pages 3-4) |
|  | 20c | Definition of analysis population relating to protocol non-adherence (eg, as randomised analysis), and any statistical methods to handle missing data (eg, multiple imputation) (not applicable ) |
| **Methods: Monitoring** | | |
| Data monitoring | 21a | Composition of data monitoring committee (DMC); summary of its role and reporting structure; statement of whether it is independent from the sponsor and competing interests; and reference to where further details about its charter can be found, if not in the protocol. Alternatively, an explanation of why a DMC is not needed (not applicable ) |
|  | 21b | Description of any interim analyses and stopping guidelines, including who will have access to these interim results and make the final decision to terminate the trial (not applicable ) |
| Harms | 22 | Plans for collecting, assessing, reporting, and managing solicited and spontaneously reported adverse events and other unintended effects of trial interventions or trial conduct (this information is reported in the ethics section on pages 3-4) |
| Auditing | 23 | Frequency and procedures for auditing trial conduct, if any, and whether the process will be independent from investigators and the sponsor (not available) |
| Ethics and dissemination | | |
| Research ethics approval | 24 | Plans for seeking research ethics committee/institutional review board (REC/IRB) approval (this information is reported in the title page) |
| Protocol amendments | 25 | Plans for communicating important protocol modifications (eg, changes to eligibility criteria, outcomes, analyses) to relevant parties (eg, investigators, REC/IRBs, trial participants, trial registries, journals, regulators) (not applicable ) |
| Consent or assent | 26a | Who will obtain informed consent or assent from potential trial participants or authorised surrogates, and how (see Item 32) (this information is reported in the ethics section on pages 4-5. Research assistants will obtain informed consent) |
|  | 26b | Additional consent provisions for collection and use of participant data and biological specimens in ancillary studies, if applicable (not applicable ) |
| Confidentiality | 27 | How personal information about potential and enrolled participants will be collected, shared, and maintained in order to protect confidentiality before, during, and after the trial (this information is reported in the ethics section on pages 4-5) |
| Declaration of interests | 28 | Financial and other competing interests for principal investigators for the overall trial and each study site (this information is reported in the declarations on page 5) |
| Access to data | 29 | Statement of who will have access to the final trial dataset, and disclosure of contractual agreements that limit such access for investigators(this information is reported in the declarations on page 5) |
| Ancillary and post-trial care | 30 | Provisions, if any, for ancillary and post-trial care, and for compensation to those who suffer harm from trial participation applicable (not applicable ) |
| Dissemination policy | 31a | Plans for investigators and sponsor to communicate trial results to participants, healthcare professionals, the public, and other relevant groups (eg, via publication, reporting in results databases, or other data sharing arrangements), including any publication restrictions applicable (not applicable ) |
|  | 31b | Authorship eligibility guidelines and any intended use of professional writers applicable (not applicable ) |
|  | 31c | Plans, if any, for granting public access to the full protocol, participant-level dataset, and statistical code applicable (not applicable ) |
| Appendices |  |  |
| Informed consent materials | 32 | Model consent form and other related documentation given to participants and authorised surrogates (not applicable ) |
| Biological specimens | 33 | Plans for collection, laboratory evaluation, and storage of biological specimens for genetic or molecular analysis in the current trial and for future use in ancillary studies, if applicable(not applicable ) |

*It is strongly recommended that this checklist be read in conjunction with the SPIRIT 2013 Explanation & Elaboration for important clarification on the items. Amendments to the protocol should be tracked and dated.
