# Supplementary material for: Feasibility of conducting a pilot randomized control trial of a psycho-education intervention in patients with a first episode psychosis in Uganda—A study protocol
Source: PLoS One. 2022 Jul 29;17(7):e0268493. doi: 10.1371/journal.pone.0268493 (PMC9337703; doi:10.1371/journal.pone.0268493)
Supplement: S2 File — (DOCX) [file pone.0268493.s003.docx]

**The effect of psycho-education on clinical outcomes among patients with a first episode psychosis in central Uganda - A pilot randomized control trial.**

**Clinical trials.gov registration number:** NCT 04602585

Makerere University School of Medicine Research and Ethics Committee IRB approval number (**Rec Ref 2020-161**)

**Methods**

**a) Eligibility criteria:** Individuals will be eligible if they have been diagnosed with a first episode psychosis (FEP) and considered (by the attending health care provider) to have demonstrable resolution of active symptoms following the use of antipsychotic medications, and deemed clinically stable for a discharge. The diagnosis of FEP will be confirmed through an interview conducted a by a research assistant (RA) using the Mini International Neuropsychiatric Instrument (M.I.N.I). Participants (adults ≥18 years) will be required to provide written informed consent and should reside within a 21 km radius from Kampala.

Exclusion criteria: Individuals will be excluded if they present with a psychosis secondary to a medical or substance use disorder.

**b) Data collection methods**

i) Training: RAs and VHTs will each undergo 4-day’s training in the study procedures.

ii) Random sequence allocation: We will use a simple random technique to randomize participants. Our biostatistician will write the words intervention, insert them in 40 opaque envelops and seal them, then do the same for controls

iii) Data collection: RAs shall identify potential participants, provide them with information about the study, obtain written informed consent, then administer the University of California, San Diego Brief Assessment of Capacity to Consent (UBACC) [[1](#_ENREF_1)] to assess participant comprehension of the consent process.

RA’s will administer a) standardized questionnaire to collect demographic and clinical variables (age, gender, address, education, date of symptom onset) b) MINI to confirm a diagnosis, c) Young Mania Rating Scale (YMRS) [[2](#_ENREF_2)] for individuals with bipolar illness or Positive and Negative Symptoms of Schizophrenia Scale (PANSS) [[3](#_ENREF_3)] for individuals with schizophrenia spectrum disorders to assess symptom severity d) World Health Organization Disability Assessment Schedule, e) Internalized Stigma of Mental Illness scale and f) a medication adherence measure

iv) Blinding participants and RA to the study arm: Participants will then pick an envelope and open it to see which study arm they belong. VHTs will deliver 6 psycho-education sessions (1 per month) to participants in intervention arm and their family members at the participant’s residence. The PI and RAs will sit in some of the sessions during the pilot phase of data collection to ensure fidelity to the manual. We will conduct 5 exit focus group discussions with patients and family members to assess acceptability to the use of the manual. Participants in the control arm will get usual treatment. RA’s will schedule participants from both arms for an assessment using the same instruments at weeks 4, 12 and 24 at the clinic

v) Data analysis plans: We will conduct an intention to treat analysis and compare the groups at baseline, weeks 4, 12 and 24. We will assess the effects of the intervention on symptom severity (PANSS or YMRS). We will assess for potential confounders, mediators and effect modifiers using generalized linear estimates.

vi) Feasibility of data collection: We anticipate getting ethical approvals and conducting formative work in 3 months, recruiting participants in 3 months and follow-up in 6 months

**c) Participant identification and recruitment:**

i) Identification: Health care workers in the different wards and out-patient departments of Butabika Hospital will be informed about the study. Trained RA shall liaise with the clinicians to identify potential participants for recruitment at the time of admission to the wards. RA’s will then assess participants who are due for discharge (patients with a clinical response to medications) for possible enrollment, and provide them with information about the study. Patients who access care at the out-patient clinic (who may not be admitted) but are eligible will also be approached by the RA for enrollment.

ii) The informed consent procedure: The RA’s will invite potential participants to take part in the study, and obtain written informed consent. During the consenting process, the purpose of the study will be described further, the procedures will be explained, and the benefits of taking part in the study will be outlined. Upon demonstrating understanding and being given a chance to ask questions, the potential participant will then provide a witnessed, signed or thumb print consent.

iii) Assessing the capacity to consent: We will administer the University of California, San Diego Brief Assessment of Capacity to Consent (UBACC) [[1](#_ENREF_1)] instrument to assess whether the participants have understood the consent process. The UBACC will be translated into Luganda, the commonly spoken local language at the study site. The UBACC is a 10-item scale comprised of 3 factors that evaluate understanding, appreciation and reasoning. It has been used in the Ugandan setting for the Neuro-GAP project, although it is yet to be culturally adapted for use in these settings. A score of less than 14.5 on three separate occasions indicates that the participant has not understood what the study is all about. Such participants will not be recruited, but will be given a chance to return at a later date for recruitment (within a week). We will record the number of participants who fail the UBACC at baseline and can’t be recruited and those who do so after being invited a week later.

We will ask participants to provide us with their demographic information (age, gender and education level) and examine whether there are significant differences between participants who are able to consent and those who are not. Participation in this study is completely voluntary. The patient has the right to withdraw at any time during the study, including at follow-up. We will document the reasons for withdrawal. Each interview is anticipated to last a minimum of 120 minutes —there will be 2-3 breaks at 40 minute intervals in between the interviews.

**Study measurements**: Trained RA will administer the following standardized questionnaires to all participants. All study questionnaires will be translated into Luganda, the commonly spoken local language at the study site.

i. Demographic and clinical parameters: We will (a) document the age, gender, physical address, contact information, marital and employment status, education level, date/month/year of onset of current illness. We will document whether the participant lives within a catchment area of ministry of health supported VHT; we will separately contact the VHT and get their details. We will also request for information from the next of kin for future contact in the event of a loss to follow-up. (b) we will document the age of onset of symptoms, duration of illness before accessing hospital care (acute if it is within 6 months of onset and chronic if it is more than 2 years), whether the participant has received prior treatment for the psychosis (traditional or faith healers), whether or not the patient had a say in the choice of antipsychotics that was prescribed to them.

ii. We will administer the UBACC [[1](#_ENREF_1)] to assess participant’s capacity to provide informed consent.

iii. The M.I.N.I 7.0.2 [[4](#_ENREF_4)] psychosis, depression, bipolar affective disorders, substance use disorder, PTSD and generalized anxiety modules will be used to confirm the presence of a psychoses, and other CMD. The MINI has been used in multiple Ugandan study settings including the Neuro-GAP project, although it is yet to be validated for use in these settings

iv. Symptom severity assessed using the YMRS or PANSS. Both the YMRS and PANSS have been used in Ugandan study settings although it is yet to be validated for use in these settings

v. The presence of medication side effects will be assessed using the modified version of the Glasgow Antipsychotic Side Scale (GASS)

vi. The World Health Organization Disability Assessment Schedule Version 2 (WHODAS 2.0) will be used to assess the level of social and occupational functioning of participants. There is limited data about the use of the WHODAS in Ugandan populations.

vii. Adherence to antipsychotics will be measured using the medication adherence rating scale (MARS) for psychosis [[5](#_ENREF_5)]

ix. We will document mortality from any causes in the participants using the WHO verbal autopsy scale.

| **Instrument** | **Baseline** | **Follow-up** |
| --- | --- | --- |
| Consent forms, UBACC(administered a day earlier) |  |  |
| Demographic parameters | Yes | No |
| UBACC | Yes | No |
| MINI | Yes | Yes |
| Physical exam | Yes | Yes |
| Symptom severity using the YMRS/PANSS | Yes | Yes |
| GASS | Yes | Yes |
| WHODAS 2.0 | No | Yes |
| Medication adherence using the MARS | No | Yes |
| WHO Audit to assess all course mortality | No | Yes |

**Pilot data collection:** We will conduct a pilot of data collection among 5 participants, and use semi-structured questionnaires to document clarity of study questionnaires, barriers to implementation and ways of circumventing the barriers.

**The intervention:** After discharge, the program manager will link the participants with a VHT nearest to them. The participants (randomized to the intervention arm) will be informed during the consent procedure that they will undergo 6 psycho-education sessions (1 per month) together with a family member at the participant’s residence. The VHTs and participants will meet and schedule appointments for the next engagements. This shall be done on a case by case basis. Some psycho-education sessions could take place in the patient’s residence, others in the nearest public space (school or church or mosque compounds). We will document where the majority of these sessions happen. This will help us document feasibility. The PI and RAs will sit in some of the sessions during the pilot phase of data collection to ensure fidelity to the manual.

**Follow-up:** Participant Follow-up: We shall collect the same data as at baseline from participants at month 3 then every six months. In the event that the participant accesses health care and we are not aware, we will review their medical records and abstract information collected during the course of routine care (where they exist). However, all attempts will be made to collect data directly from the participants at all times.

Medical Record Review: There is a possibility that participants in the control arm will return to access care at the facility and be missed by the RA. They could also be admitted to the facility due to other health complications. We will review participant’ medical charts and abstract information about any admission to the hospital, duration of stay, laboratory parameters, and any other recorded complications.

**Adverse event reporting during participant follow up:** We anticipate that this project will have minimal adverse events that are directly related to the study. However, in the event that we observe any adverse events as a result of the use of prescribed medications during clinical care or loss of privacy/confidentiality, then we will report it promptly to the relevant regulatory bodies per requirement. Confidentiality could be broken in the event that the RA gets information related to the following: a) participant is suicidal, b) participant threatens to commit a homicide, and c) participants reports a sexual abuse to themselves or other parties. The RA will immediately inform the PI about such, and appropriate action will be taken including reporting such cases to the administration of Butabika Hospital.

Potential risks: There is a potential risk of developing severe psychological distress during the interviews as a result of answering questions that are deemed private by the participant. RA will be trained to identify any of such distress, and the interview will be terminated. Participants may be asked to continue with the interview only if they feel like doing so. Such adversities will be reported to the PI and SOMREC. There is also a risk of having information about participants made available in the public domain. We will guard against this by having all identifying information of the patients locked away in file cabinets and password locked computers. The risk to loss of information is minimal.

Benefits: There are no direct monetary benefits to be gained by the individuals. However, participants will receive regular assessments for their symptoms for the study period. Those in the intervention arm will receive psycho-education sessions. Furthermore, participating in this study will also help scientists to learn more about what predicts relapse in patients with psychosis

Community Tracing: In the event that a participant has not appeared for a clinic visit on their scheduled clinic appointment, we will contact them or their appointed person through telephone. If neither the patient nor the contact person can be reached by phone three months from the last date of their scheduled appointment, we will make active attempts to trace the participant at their residence by liaising with the VHT based in the same location. In the event that the participant can’t be traced at their place of residence, we will consider them as a potential loss to follow-up. There exists a number of village health team members who provide care to non-mental health clients. We will utilize their knowledge about the village to identify individuals in the cohort.

**Data analysis plans:** An intention-to-treat analysis will be conducted to compare the groups at baseline and within 4, 12 and 24 weeks to assess the effects of the intervention on symptom severity and retention in care. The dependent variable (severity of symptoms measured using the PANSS/YMRS) will be calculated as a continuous variable. Independent variables including adherence and retention in care will be presented as continuous and categorical variables.

Baseline characteristics of the intervention and control arms will be compared at the 5% level to assess if successful randomization was achieved. Data on potential confounders and effect modifiers, including variables that fail to achieve successful randomization (e.g. socio-demographic parameters) shall be used to control for confounding and effect modification. Between-subject analysis at week 24 will be used to assess the direct effect of the intervention by determining if there is a significant difference between the mean MHL scores in the intervention and control arms. Within-subject analysis will be performed among patients in the intervention arm by applying the Generalized Estimating Equations method on repeated measures data

**References**

1. Jeste D.V, Palmer B.W, Appelbaum P.S, Golshan S, Glorioso D, et al. (2007) A new brief instrument for assessing decisional capacity for clinical research. Arch Gen Psychiatry 64: 966-974.

2. Young R.C, Biggs J.T, Ziegler V.E, Meyer D.A (1978) A rating scale for mania: reliability, validity and sensitivity. Br J Psychiatry 133: 429-435.

3. Stanley R. Kay, Abraham Flszbeln, Lewis A. QpJer (1987) The Positive and Negative Syndrome Scale (PANSS) for Schizophrenia. Schizophrenia Bulletin 13: 282-287.

4. Sheehan .D.V, Lecrubier Y, Harnett-Sheehan K (1998) The Mini International Neuropsychiatric Interview (M.I.N.I.): The Development and Validation of a Structured Diagnostic Psychiatric Interview. . J Clin Psychiatry 59: 22-23.

5. Thompson K, Kulkarni J, Sergejew A.A (2000) Reliability and validity of a new Medication Adherence Rating Scale (MARS) for the psychoses. Schizophr Res 5: 3.
