# Supplementary material for: Feasibility of conducting a pilot randomized control trial of a psycho-education intervention in patients with a first episode psychosis in Uganda—A study protocol
Source: PLoS One. 2022 Jul 29;17(7):e0268493. doi: 10.1371/journal.pone.0268493 (PMC9337703; doi:10.1371/journal.pone.0268493)
Supplement: S1 File — (PDF) [file pone.0268493.s002.pdf]

Psycho-education for persons with Psychosis

The information contained in this booklet is meant to provide a brief about

1. The signs and symptoms of psychosis (the abnormal experiences that you went through is),
2. The challenges that individuals with psychosis go through,
3. The causes of these psychosis,
4. The treatment options for these experiences,
5. The reasons for treating them and
6. Some of the myths around this condition

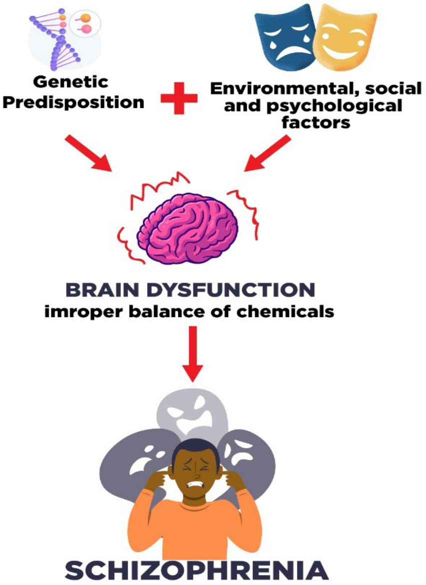

1. What is Psychosis? What are the Signs and Symptoms of Psychosis?

Description

Psychosis is a mental condition, which when we get it, interferes with how we think, as well as how we see and interpret things around us, making it difficult for us to decide and know what is real and what is not real. These experiences are not nice and they make us uncomfortable. In addition, they may be scary and confusing to us.

The signs and symptoms of psychosis may include

a) Disturbances in how we see and understand things around us.

Some people, when they get *psychosis*, may see, hear, feel, smell, and taste things that the other people around them don't not. For example, they can hear voices of people that

© Dickens Akena 2020-MAK-RIF. MAK/DVCFA/113/20

cannot be seen by anyone, talking to them, yet people sitting with them do not hear. Such voices of unseen people may order (or command) the person to do things for example, “Go walk around the village!.” The voices may also talk about everything the person does (often in a negative way). At times, the voices may discuss or gossip about the person. The affected person may recognize whose voices they are, but most times they are not sure exactly whose they are nor what they want. Sometimes individuals who are hearing voices may respond to them aloud. People with psychosis also sometimes see things that others around them don’t see, or they may smell things others don’t smell. These experiences that disturb how a person sees and interprets things around them are called *hallucinations*.

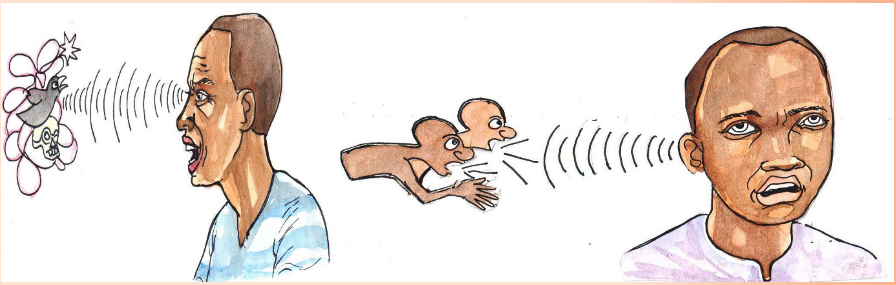

b) Disturbance in thinking.

In some instances, there is a disturbance in one’s thinking and beliefs. For example, some people may believe that their life is in danger and this threat comes from either those they know, or unknown people or entities (like a community, an institution or even a government). They may try to run away from these people who want to harm them. However, in some instances, they may confront the people or persons or entities that they believe mean them harm. Please note that the individuals who are considered as persecutors may not even be aware of this fact.

c) Control by external powers.

Some individuals may believe that they are being controlled by external powers such as ancestral spirits, the devil or God. On some occasions, they may respond to the demands of these unseen powers. The people around them find these explanations or behaviors strange and difficult to believe.

d) Disturbance of mood.

In some types of psychosis (bipolar disorder in manic phases), individuals may feel excessively happy (happier than usual), together with a feeling of increased self-importance and greatness. They may have increased energy and less need to sleep (they wake up early and feel rested even after just a few hours).

Such individuals may believe that they have special powers to perform tasks that others can't perform (like miracles to heal the sick). They may also think very fast and about doing very many things at the same time (but they hardly complete these things). They often talk in a loud voice and may talk for many hours without stopping (even to strangers). When individuals are in this happy state, they may spend a lot of their money on buying things that they may not even need, and they may give out their property to strangers without carefully thinking about it. They may also have an increased desire to have reckless sex and other high risk activities. Please note that these individuals may instead of being in a good happy mood, be extremely easy to anger (irritable), especially if their plans are not fully accepted by those around them.

In summary:

The experiences of *psychosis* are real and scary to the person who has them. They are not imagined. The affected person is often confused about which experiences of their lives are real and which are not.

In the next session, we will take a look at the challenges that individuals with *psychosis* experience in their day-to-day interactions with people.

2. What are the Challenges that Individuals with Psychosis Experiences?

Individuals with psychosis have difficulty in many areas of life including social interaction, work study, taking care of themselves or their families. So how does this come about?

Difficulty in being understood when talking about their experiences:

One of the greatest challenges that individuals with psychosis experience is the difficulty in explaining their experiences to those around them. After all, those around them don't see the persons being referred to or hear what the other person is hearing or experience the control by an external force. Instead, to those around them, it appears as if the individual is talking to unseen people or making up stories or even wrongly accusing people. Remember we said in the previous session that these are real and vivid experiences to the affected person. This inability to get the people around them to appreciate their concerns is a potential source of frustration, anger, irritability and inevitable conflict.

Negative impact on social and occupational functioning:

Someone who believes that they are being spied upon, or that people are out to harm them may decide to stay indoors; staying indoors could lead to loss of their job or failure to look for one, or dropping out of school or inability to take care of their families. Some may give up school or their jobs because of a belief that there is something better or that something bad is going to happen if they continue working or studying. In some instances

individuals with psychosis may behave in socially unacceptable ways such as getting easily angered or extremely inpatient. They may attack their perceived persecutors and end up committing a crime or get harmed in the process. Similarly, a person who hears voices telling them to go perform an act such as deliver a special message may walk for many miles and could get in to trouble with a mob suspecting them of being a thief.

Difficulty in completing mental tasks (cognitive function):

cognition refers to every process by which individuals obtain and utilize knowledge / it encompasses processes such as thinking, reasoning, recognition, labelling, analysis, categorization, and planning through which people understand their environment and fit into it. Psychosis affects the way one can plan or carefully think through activities that require concentration, making it difficult for one to complete mental tasks. Such difficulties affect the ability to look for jobs or maintain them or complete school or other important areas of daily living such as self-care. Please note that such disturbance is not necessarily equal to poor intelligence. People can be intelligent, but if the processes of thinking, reasoning and planning are affected by the psychosis then they are unlikely to execute a task that they would have easily performed had they not had psychosis. Please note that psychosis is not known to directly affect or lower one's intelligence.

Stigma and social withdrawal:

A number of individuals with psychosis keep to themselves and often withdraw from social activities. Moreover, they struggle with self-stigma and stigma from the society. They may not be comfortable interacting with people out of their families and some may not even get the so much needed support from their families. The stigma and social withdrawal limits one's ability to achieve their goals in life.

In summary:

Psychotic experiences negatively affect social and occupational functioning of the individual leading to loss of opportunities in life.

In the next session, we will take a look at some of the potential causes of psychosis.

3. So, what causes Psychosis (abnormal experiences and behavior)?

The exact cause of psychosis remains unknown, however, there are a number of reasons that have associated to its development.

Imbalances in brain chemicals:

The signs and symptoms of abnormal experiences and behavior can be explained by

imbalances in some brain chemicals that control the way we think and perceive things around us. Some of the chemicals become less while others increase in amounts that are in excess of what the body requires.

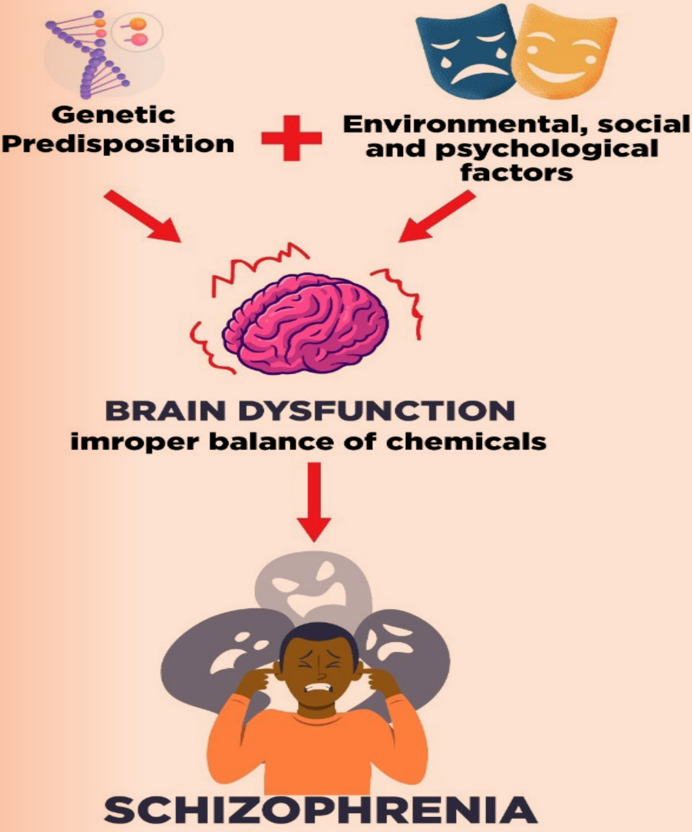

So one may ask, what causes these imbalances of the brain chemicals?

The causes of these imbalances remain unknown to a large extent, the same way it is for other chronic medical illnesses. As an example, an imbalance in insulin could lead to development of diabetes mellitus. Similarly, cancers are a result in an imbalance in the way our body cells multiply. The exact reasons as to why some people get these imbalances and others don't is not fully known. The exact cause of psychosis is not known, however, multiple factors including genetics, medical diseases or exposure to psychological stress can contribute to their development. It is important to note that there are also factors that contribute to development of chronic diseases; for example, excessive intake of sugars in someone who is genetically predisposed could lead them develop diabetes. Smoking cigarettes could also lead to development of lung cancers,

while excessive intake of fatty foods could cause heart problems. The same can happen in the development of abnormal experiences and behaviors.

Stress and substances of abuse:

Extreme amounts of psychological stress can trigger the development of abnormal experiences and behaviors in some individuals. Whereas stress in itself may not cause psychosis, its presence makes abnormal experiences and behaviors symptoms worse. Stress also makes it difficult for one to cope with the challenges of having abnormal experiences and behaviors.
`

Those who engage in the use of drugs of abuse particularly cannabis, alcohol, cocaine and many other addictive substances may also lead to the development of psychosis as well as untreated medical illnesses such as HIV

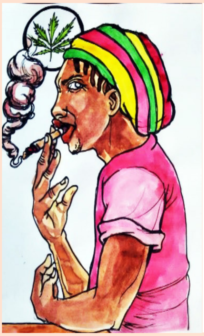

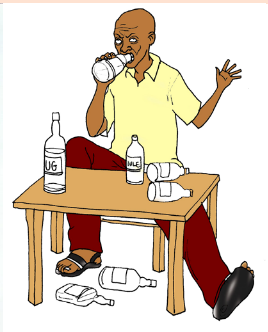

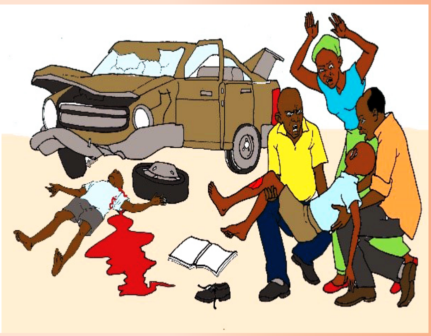

Individuals who are severely traumatized, especially in childhood may also develop psychosis.

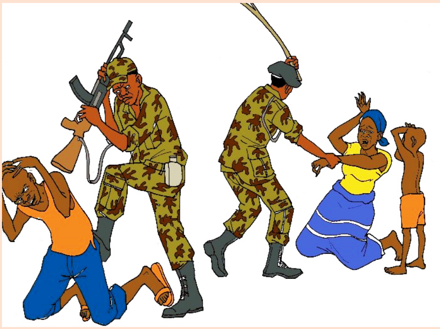

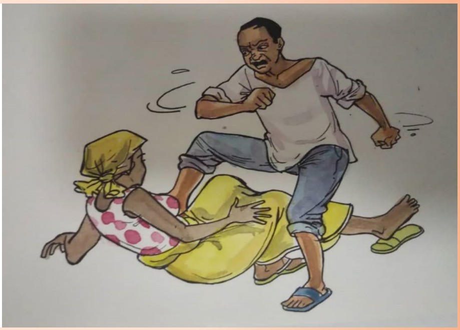

But isn't abnormal experiences and behaviors a result of spirits or evil forces? After all, affected individuals are often observed to talk to unseen people or communicate with their dead ancestors! Indeed, those who are religious may attribute their experiences to the devil while those who are not may attribute them to other worldly forces they

are familiar with. In rare occasions these experiences can be completely out of context (beliefs). Someone could say their brain is fused with the moon or that their hand is a fish.

In summary: The experiences these individuals go through are a distortion of reality and there is no known single cause of abnormal experiences and behaviors. A combination of genes and other factors could be responsible. Most times, we are completely unaware of the factors that can lead to the development of abnormal experiences and behaviors and may not be able to prevent them (apart from use of substances of course).

In the next session, we will talk about the treatment of psychosis

4. Can psychosis be treated?

Yes! We treat it using antipsychotic medications and other forms of treatment like talk therapies (psychotherapies) and social support for the patient and their family members.

What do antipsychotics medications do?

Remember we said that psychosis results from an imbalance in some brain chemicals. Antipsychotic medications reverse these imbalances and restore a balance that is needed for one's brain to properly function. When one takes the medicine as prescribed and the balance in the brain is restored, there will be a reduction in the severity of symptoms and one's mental health improves. In some instances, the person becomes symptom free after taking medications over a long time.

Does it mean that once a person is symptom free they stop taking medications?

Not at all; it is a huge mistake to stop taking medications once a person is symptom free. Please note that the chemical imbalances don't develop overnight; reversing and restoring them takes an equally long time. Psychosis in many instances is a chronic illness and the treatment is similar to the way we treat chronic illnesses like diabetes mellitus or hypertension; individuals have to take medications consistently for a long time so that they are free of the symptoms

Do these medications lead to complete recovery?

In some people, the use of antipsychotic medications leads to complete recovery. However, the majority of individuals will need to take them consistently in order for them to be symptom free. This is common especially in those who have had a long history of untreated psychosis. The earlier psychosis is treated, the better the outcomes (higher chances of complete recovery). Please note that the use of antipsychotic medications is likely to improve the chances that someone will perform their daily activities with minimal

supervision and are able to live to the best of their potential. Indeed, many people who take antipsychotics are able to work and lead fruitful and productive lives. We advise that people don't discontinue treatment on their own.

But these medications have a lot of side effects!

Yes, just like other medications, antipsychotics do have side effects. Please ask your health care provider about side effects and how you can treat or avoid them. If the side effects become intolerable, then one has to engage with their health care worker for the next course of action which may include dose adjustment or change of medication to another type.

What if I want to combine medications with other treatments given to me by my healer?

We discourage the use of herbal remedies while taking antipsychotic medications or any other medications that has been prescribed by your health care provider. Some of these herbal remedies have active chemical ingredients that can interact with antipsychotics making them weaker or even stronger and potentially dangerous!

What are the other treatments?

We have a number of talk therapies that we provide to patients who are taking antipsychotics. Most of them are geared towards provision of correct information about the disease, means of coping with stress and how to challenge the negative thoughts that come along with having a psychotic illness. A combination of medications and talk therapy is better than just one of those in isolation. We recommend it for routine care.

Patients and their family members can also be supported to connect with people or organizations that can help to engage the patient in work or other forms of social and economic support

5. Why should we treat psychosis?

Beyond achieving symptom relief, there are other reasons as to why we treat psychosis.

Individuals:

Research shows that individuals with psychosis are more likely (than those without) to get a number of diseases like HIV, non-communicable diseases like high blood pressure, and diabetes. Individuals with psychosis are also more exposed to getting road traffic accidents.

Untreated psychosis has been shown to reduce one's lifespan by up to 10 years (individuals die 10 years earlier than those without psychosis). Fortunately, treatment of psychosis has been shown to reverse these dangers. Therefore, treating psychosis leads

to benefits that go far beyond achieving symptom lessening including the improvement of one's overall quality of life and prevention of early death from the other factors.

Families:

The symptoms of severe psychosis are plain and can easily be seen by people inside and outside of one's family. The limited knowledge about this psychosis by members of the community are a major driver of stigma against the person suffering from this illness as well as their relatives. A number of families go to great lengths to hide the fact that their loved one has a psychotic illness by chaining or locking them up to keep them up in rooms. Other families are known to disown individuals with psychosis. It is not uncommon for the human rights of individuals with psychosis to be grossly abused in such situations.

Research also shows that family members living with one of their own that has psychosis undergo enormous stress and uncertainties, exasperation and despair; they ask questions and can't get answers. For example, they struggle to figure out whether their loved ones will ever achieve an independent life or end up on the streets eating from dustbins. They have difficulty trying to figure out why their loved one developed this illness. Some family members blame themselves for the illness of their loved ones. These stressful situations at times break up families. Therefore, treating psychosis leads not just to symptom relief in the patient, but to improvement in the way the family functions

Communities:

When a breadwinner (the head of the household) develops psychosis, then the members of that family get into a lot of difficulties in meeting the basic needs like food, rent school fees and medical care. A productive person who becomes psychotic may disrupt peace and social functioning in society and in some occasions; can destroy property and cause insecurity. Remember that individuals with untreated psychosis may not be able to contribute significantly to the development of their society, they are unlikely to make money or pay taxes, yet they still have to access medical and essential services. The responsibility for the members of such a family shifts to the community because of the very nature of our society (we take care of those who cannot take care of themselves).

In summary: The burden of psychosis is not limited to the person. The burden of psychosis is shared by not just the family members but the community and ultimately the state. We need to do everything within our means to make sure that individuals with psychosis receive treatment.

In the next session, we will talk about the myths associated with psychosis

6. Myths...true or false?

Is psychosis contagious? Can it be passed on to others? False!

Psychosis is not a contagious disease like Ebola or COVID-19. In some cases though, people can inherit the genes that make them more likely to develop psychosis. However, this genetic link is not as direct as that seen for conditions like sickle cell disease or albinism (where if both parents are carry the gene, then their children have a high likelihood of getting the condition). Earlier on, we hinted on how passing on the genes for psychosis from parents follows a complex pattern; many people who have psychosis don't have an immediate family member with psychosis.

Not everyone who takes medication becomes symptom free. True!

In some individuals, symptoms of psychosis may persist even with treatment. However, the use of antipsychotics certainly reduces the severity of symptoms and allows the person to be able to do activities and work. If symptoms persist, first ensure that you are taking the medications as the health care provider told you and not missing doses, then consult your health care provider. They may arrange for other forms of talk therapies that may need to be combined with medication.

Do antipsychotics lead to permanent brain damage and inability to have children? Not directly!

Whereas we can never know all the side effects of medications, permanent brain damage or inability to have children as a direct result of use of antipsychotics is rare.

Are antipsychotic medications addictive? No!

Some people think antipsychotics are addictive and that once you take them for a long time you will never leave them. Whereas some medications that are used in treating other mental illnesses may be addictive, antipsychotics per-se are not addictive. We usually encourage that the person with psychosis engages their health care worker if they have any queries.

Can we talk to people with psychosis and get them to 'abandon' their belief? No!

It is not possible to talk people out of their psychotic beliefs or perceptual disturbances. You will recall that in the earlier sessions we said that the experience of psychosis is real to the person experiencing it. Actually trying to dismiss this narrative is a potential source of conflict. Psychotic symptoms will go away only after the chemical imbalances have been restored through the use of medications. However, in talk therapies, the patient is helped to learn how to deal with these psychotic experiences and learn to identify what is real and unreal

In summary:

a) Psychotic experiences are real and frightening situations to the affected person and not an imagination. The affected person is often torn between what is real and what is not.

b) Psychotic experiences negatively affect how one interacts with others and working ability leading to loss of opportunities in life.

c) There is no known single cause of psychosis. A combination of genetics and other factors could be responsible.

d) Psychosis is treated using antipsychotics which leads to symptom relief and a return to functioning

e) The burden of psychosis is not limited to the person suffering from it but family members, the community and ultimately the state. We need to do everything within our means to make sure that individuals with psychosis receive treatment.

© Dickens Akena 2020-MAK-RIF. MAK/DVCFA/113/20
